# Modulatory Effects of *Urtica dioica* on Neurodegenerative Diseases: Unveiling the Latest Findings and Applications Related to Neuroinflammation, Oxidative Stress, and Cognitive Dysfunction

**DOI:** 10.3390/antiox14070854

**Published:** 2025-07-12

**Authors:** Ahlem Chira, Stefano Lorenzetti

**Affiliations:** 1 Laboratory of Biomathematics LR22ES01, Faculty of Sciences of Sfax, BP 1171, Sfax 3000, Tunisia; chiraahlem@gmail.com; 2 Department of Food Safety, Nutrition and Veterinary Public Health, Italian National Institute of Health, I-00161 Rome, Italy

**Keywords:** *Urtica dioica*, neurodegenerative diseases, phytochemical, neuroprotection, neuroinflammation, oxidative stress, cognitive dysfunction, animal models

## Abstract

Over the past decade, *Urtica dioica* L. (*U. dioica*) has gained prominence in biomedical research, particularly for its potential therapeutic applications in neurodegenerative diseases. This comprehensive review explores its botanical characteristics, toxicological considerations, and extensive traditional medicinal uses. Emphasizing the roles of phytochemical constituents such as flavonoids and overall polyphenolic compounds, this review examines their impact on mitigating critical pathways, such as neuroinflammation, oxidative stress, and mitochondrial dysfunction—all of which are implicated in Alzheimer’s Disease (AD), Parkinson’s Disease (PD), and Multiple Sclerosis (MS)—and, overall, in neurodegenerative processes in both humans and animal models. Notably, some phytochemicals are known to modulate crucial pathways for neuronal plasticity, learning, and memory, thereby enhancing cognitive functions. Hence, the potential of *U. dioica*-based therapies to improve cognitive function and pave the way for future therapeutic developments in neuroprotection is underscored.

## 1. Introduction

Across the century, neurodegenerative diseases such as Alzheimer’s Disease (AD), Parkinson’s Disease (PD), and Amyotrophic Lateral Sclerosis (ALS) have posed enduring challenges due to their complex etiology and profound impact on neuronal function [1]. These disorders are characterized by the progressive degeneration of neurons, leading to cognitive decline, motor impairment, and ultimately, significant disability [2]. A common hallmark is neuronal disruption driven by metabolic failure, inflammation, and protein misfolding [3]. For example, mitochondria, which are crucial for energy synthesis, metabolite oxidation, and cellular homeostasis, which play pivotal roles in maintaining neuronal health [4]. Moreover, studies indicate that interleukin-6 (IL-6) can promote Amyloid-beta (Aβ) clearance in AD models through reactive gliosis, suggesting complex interactions between inflammation and disease pathology [5]. Despite decades of intensive research, effective treatments for these diseases remain elusive, highlighting the urgent need for innovative therapeutic strategies that can address their multifaceted pathophysiology. In this context, the use of neuroprotective medicinal plants from alternative medicines has gained prominence [6]. Neuroprotective medicinal plants have the potential to repair brain damage associated with neurodegeneration and enhance learning and memory by promoting the development of new synapses [7]. *Urtica dioica* L. (*U. dioica)*, a perennial plant in the Urticaceae family, has been used in folk medicine for centuries and across continents [8,9]. Its rich phytochemical composition has been shown to be effective in cellular and molecular systems related to neurodegenerative diseases, suggesting promising prospects for disease prognosis [10]. Studies suggest that *U. dioica* enhances the synthesis of trophic factors in the brain, which is associated with improved learning, memory, and long-term potentiation [11]. Additionally, *U. dioica* supplementation has been shown to promote neurogenesis, enhance brain plasticity, increase resilience to brain damage, and improve cognitive and memory performance [12]. To date, no comprehensive review has been published regarding the current state of *U. dioica*-based remedies for neuroprotection. The current review aims to provide an updated overview of existing neurodegenerative diseases and the potential role of *U. dioica* in combating them. This goal can be achieved by considering animal models and discussing recent advancements in experimental design. In fact, increasing evidence has shown that *U. dioica* can improve memory and reduce chronic stress-related dysfunctions of the central nervous system (CNS) in animal models [13]. Despite this promising evidence, exploration in this field is relatively recent, making it an appealing option for medicinal chemists to further investigate.

Evidence from studies published between 2015 and 2025 is incorporated into this review. The terms “*Urtica dioica*,” “neurodegenerative diseases,” “oxidative stress,” “mitochondrial dysfunction,” and “neuroinflammation” were used in a systematic search on PubMed, Scopus, and Web of Science to find relevant publications. Peer-reviewed publications providing experimental and/or in vivo and/or mechanistic findings were the only ones that were accepted. Only when necessary to provide a summary of more general ideas, review articles, and meta-analyses were cited.

## 2. Urtica dioica

### 2.1. Botanical Study

**Figure 1:**
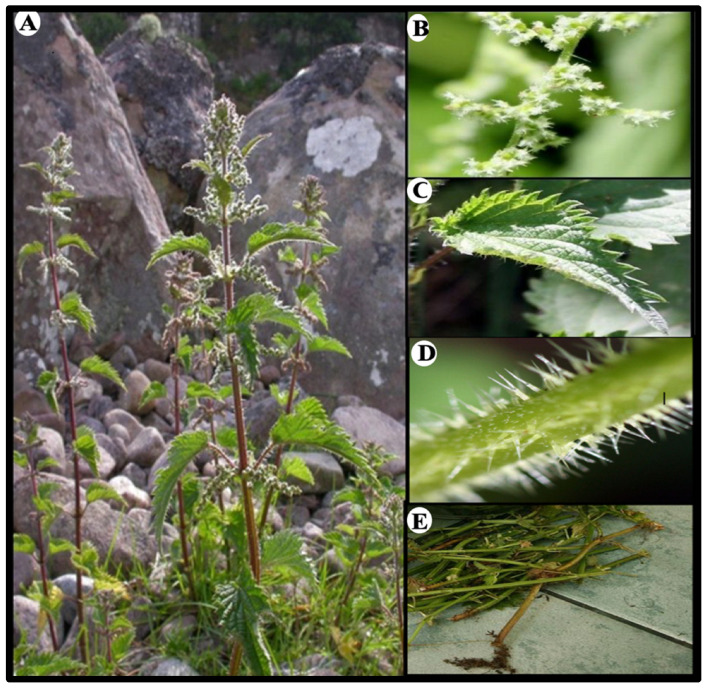
Parts of the plant *Urtica dioica*: (**A**) *Urtica dioica* whole plant, (**B**) flowers, (**C**) leaves, (**D**) stinging hairs, and (**E**) roots.

*U. dioica*, commonly known as the “stinging nettle” (Figure 1), is a perennial herb belonging to the Urticaceae family [8]. This herbaceous plant typically grows to a height of 1 to 2 m and possesses rhizomes. It falls under the Rosales order and is characterized by the presence of stinging cells in the genus *Urtica* [14]. The erect stems of *U. dioica* are strong, hairy, usually unbranched, and quadrangular in shape [15]. Young plants exhibit green leaves, whereas mature plants develop purple or reddish foliage [16]. The opposite leaves are elongated and egg-shaped, with a distinctly serrated edge and pointed tip [17].

Both the leaves and stems are densely covered in stinging hairs, many of which contain sharp points that release a stinging liquid upon contact [18]. The flowering period of *U. dioica* typically occurs from July to August in temperate regions, although it may vary depending on geographic location and climate. The species is dioecious, meaning it has separate male and female plants, which is a consistent trait across its range [19]. The small, unisexual flowers are clustered in groups on slender, branching spikes that emerge from the leaf axils. With their greenish coloration, female flowers feature a single ovary that contains one style and a brush-like stigma [20]. Male flowers possess four elongated, flexible stamens that are curved inwards in their bud stage and have a yellowish hue [14]. Following pollination, stinging *U. dioica* produces oval-shaped achenes, which are one-seeded fruits predominantly black or dark brown in colour. The root system consists of a taproot that branches into fine rootlets, enabling *U. dioica* to spread and thrive [21].

### 2.2. Toxicological Studies

Numerous toxicological studies have been undertaken to evaluate the toxicity profile of this plant, and the findings consistently indicate that its utilization is linked to minimal to negligible harm. Dar et al. [22] utilized the well-established *Artemia salina (A. salina)* technique to evaluate the toxicity of an extract derived from *U. dioica*. The results of the investigation showed that when utilized against *A. salina* larvae, both the aqueous extract and the herbal formulation termed Hexane Fraction-2 (HF2) of *U. dioica* offered exceptionally large margins of safety with a 50% lethal concentration (LC50) > 1000 μg/mL. The two crustacean species *A. salina*, as a toxicity assay model, presents some practical advantages for use at a preliminary stage, but the interpretation should be verified under some conditions [23]. It’s dependent on salinity, pH, oxygen content, and solvent concentration, and all these parameters need to be precisely controlled to have a reliable and consistent output [24]. The variation in LC50 results makes it clear that we need to be careful when applying data from *A. salina* to other species [25]. For risk assessments to be meaningful, toxicity estimates have to be both accurate and consistent [26]. By understanding what drives this variability, we can design better tests and make more informed interpretations of ecotoxicological data [27].

Dar et al. [22] also performed an acute toxicity test in *Wistar* rats, and the findings indicated no mortality after 24-hour of treatment with aqueous and hexane extracts of *U. dioica*, indicating that this herbal medication has sufficient safety potential.

Tekin et al. [28] conducted an acute toxicity investigation using the fixed oil of *U. dioica* in rodent models. The oil was administered in doses ranging from 0.2 to 12.8 mL/kg body weight (bw) *intraperitoneally* (*i.p.*), consistent with the liquid nature of the extract. No mortality occurred within the 72-hour observation period, even at the maximum dose of 12.8 mL/kg bw *i.p.*, confirming its non-toxic profile within the tested range.

In zebrafish larval models [29], *U. dioica* ethanolic extract (UDE) demonstrated a safe profile when it was administered at a concentration of 25 mg/L in E3 medium. This concentration, applied in both preventive and curative treatment protocols, caused no observable toxic effects or mortality in the control groups. Additionally, UDE exhibited significant protective effects against chlorpyrifos-induced toxicity, reducing teratogenic effects, preserving telencephalon morphology, and mitigating abnormal locomotor behavior. Overall, these observations reinforce the potential of the extract for therapeutic applications in mitigating environmental toxicity.

Nencu et al. [30] conducted a study using rabbit models to assess the potential hazards associated with this herb by administering 50 mL of a 50% UDE for a duration of 10 days. Occasional instances of diarrhea were observed. However, subcutaneous injections with doses ranging from 5 to 20 mL showed varying tolerability, with chronic injections leading to significant weight loss and mortality in some cases. The authors determined that the intravenous fatal dosage of extract is 1.5 mL at fivefold greater concentrations and that heating the extract reduces its toxicity. The major symptoms displayed by the animals under research were increased breathing and central excitatory behaviour.

### 2.3. Traditional Uses

*U. dioica* is widely distributed across South Asia and the Indian subcontinent and has a rich medicinal heritage [31]. Indeed, it is widely recognized as a significant source of bioactive compounds (Table 1) with pharmacological relevance [32]. Ethnopharmacologically, it is esteemed for its therapeutic properties and is known to both prevent and treat various illnesses [33]. Traditionally, it has been utilized to address conditions such as hypertension and hepatic disorders [34], although a comprehensive study of its pharmacological profile and chemical constituents has only been undertaken recently [35]. Medicinally, it is employed for treating ailments such as arthritis, allergies, urinary tract issues, and skin disorders [36]. Additionally, in nutritional contexts, *U. dioica* has been applied in the food and pharmaceutical industries as a source of chlorophyll, which is utilized as a food coloring ingredient (E140) [37].

### 2.4. The Roles of Phytochemical Compounds of Urtica dioica in Neurodegenerative Diseases

Several studies have looked into the therapeutic potential of *U. dioica* in the context of neurodegenerative diseases [43]. Instead of examining isolated compounds on their own, it makes more sense to consider the plant as a whole—as a complete extract where various nutrients and phytochemicals may work together [44]. In practice, the compounds found in *U. dioica* are probably not acting in isolation [45]. More likely, there’s some level of interaction between them—one affecting how the other is taken up, processed, or even stabilized in the body [46]. That might be part of the reason why it’s difficult to link the observed effects to a single constituent [47]. The response seems to reflect the system as a whole, not any one molecule alone [48]. The cumulative response is more plausibly the result of coordinated actions within the plant’s phytochemical matrix [48]. These interactions—which reflect the full complexity of the plant—are outlined in more detail in Table 2 and Table 3, which summarize some of the key antioxidant, anti-inflammatory, and neuro-supportive effects reported so far.

## 3. Neuroinflammation, Oxidative Stress, and Mitochondrial Dysfunction in Neurodegenerative Diseases

Alzheimer’s and Parkinson’s diseases as well as ALS are all characterized by common molecular abnormalities, most notably chronic neuroinflammation, oxidative stress, and mitochondrial dysfunction [5]. These interrelated pathways lead to gradual neuronal death and cognitive decline, and they have been seen in both clinical and experimental research [83]. Microglia are the resident immune cells of the central nervous system and play a major role in neuroinflammation [84]. When triggered by infections or injury, they become active and release pro-inflammatory molecules [85]. Nuclear Factor kappa B (NF-κB) is one of the main pathways involved in this process, regulating genes linked to inflammation [86]. It has a dual role: in neurons, it supports survival and synaptic function, but in microglia, its activation contributes to chronic inflammation and neurodegeneration [87]. Some of the compounds found in *U. dioica*, like tannins and chlorogenic acid, seem to play a role in how microglia behave during inflammation [88,89]. These plant-derived compounds might help by keeping microglial activation under control, which could, in turn, ease the pressure that chronic inflammation puts on the nervous system [90].

**Figure 2:**
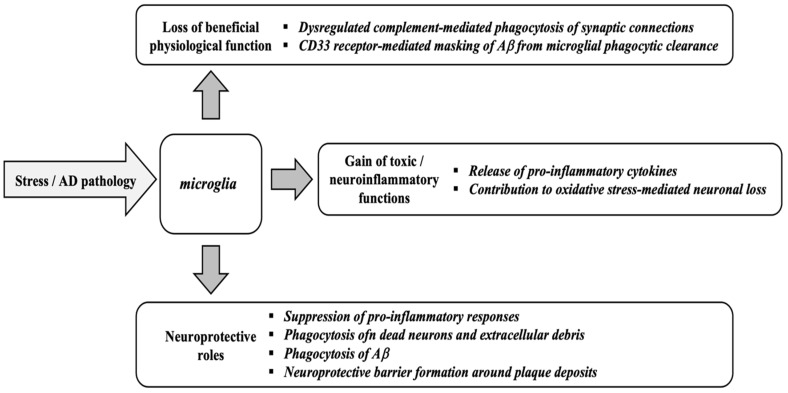
Microglial functions in response to stress and Alzheimer’s disease (AD) pathology. Adapted from [98].

As described in Figure 2, microglial activation plays an important role in Alzheimer’s disease, regulating plaque dynamics and immunological signaling. These cells exhibit diverse functions such as phagocytosis, chemotaxis, and activation, which impact plaque dynamics and coverage [91]. The complex interactions among microglia, β-amyloid protein, and neurofibrillary tau tangles in AD underscore the importance of understanding microglial responses to develop effective therapeutic strategies [92]. Signaling cascades, including Nuclear factor erythroid 2-Related Factor 2 (Nrf2)-Antioxidant Response Element (ARE) pathway and Mitogen-Activated Protein Kinase (MAPK)-Extracellular signal-Regulated Kinase (ERK), which are already dysregulated in Alzheimer’s disease, lead to prolonged neuroinflammation and neuronal stress [93].

Neuroinflammation also affects signaling pathways involved in mood, behavior, and stress-related disorders [94]. Elevated levels of inflammatory mediators in the brain have been correlated with impaired neurological function in both clinical studies and animal experiments [95]. This inflammatory state has also been found to reduce neural plasticity, hampering the ability of the brain to adapt and reorganize [96]. Reducing brain inflammation has been shown to improve related behavioral impairments [97].

**Figure 3:**
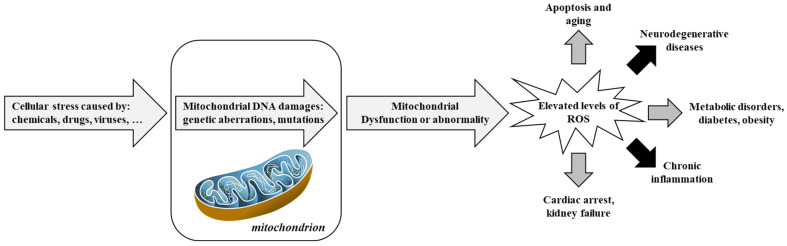
Involvement of mitochondria in oxidative stress and diseases. Adapted from [100]. Reactive Oxygen Species, ROS.

**Figure 4:**
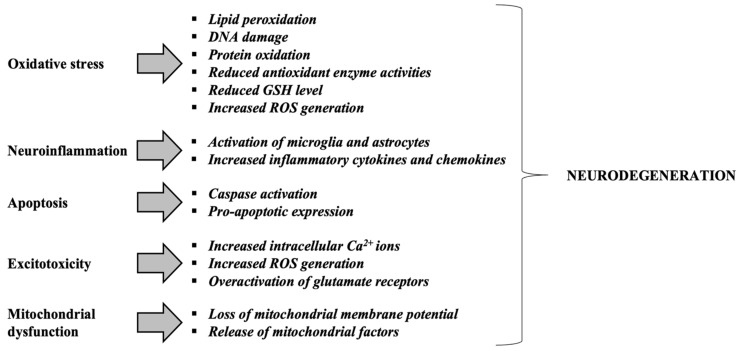
Mechanisms contributing to neurodegeneration in neurodegenerative disorders. Adapted from [101]. Glutathione or γ-glutamyl-cysteinyl-glycine, GSH; Reactive Oxygen Species, ROS.

Figure 3 and Figure 4 provides an integrative view of converging mechanisms that drive neurodegeneration, including oxidative stress, protein aggregation, neuroinflammation, excitotoxicity, mitochondrial dysfunction, and apoptosis. These interconnected pathways—each detailed in preceding sections—collectively contribute to progressive neuronal loss in disorders such as Alzheimer’s and Parkinson’s diseases [99].

**Figure 5:**
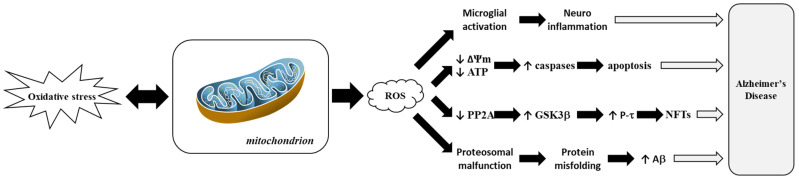
Representation of mitochondrial dysfunction induced by Reactive Oxygen Species (ROS) in Alzheimer’s disease. Adapted from [103]. Amyloid beta-peptide or amyloid-beta, Aβ; Adenosine TriPhosphate, ATP; Glycogen Synthase Kinase 3 beta, GSK3β; Mitochondrial Membrane Potential or delta psi membrane, ΔΨm; NeuroFibrillary Tangle, NFT; Protein Phosphatase 2A, PP2A; tau Protein, P-τ.

Figure 5 highlights how ROS-driven mitochondrial dysfunction contributes to AD progression. This process is closely linked to hallmark features of AD, such as amyloid-β (Aβ) plaque accumulation and tau hyperphosphorylation, which correlate with cognitive decline [102]. Disturbances in tau and other cytoskeletal proteins also play significant roles in AD. Microtubule-Associated Protein 2 (MAP2), which is crucial for neuronal maintenance and plasticity, shows altered expression levels in AD [103]. Phosphorylation events involving MAPK3, which is pivotal in neurogenesis, and Amyloid Precursor Protein (APP) processing, are dysregulated in AD [104]. In transgenic mouse models, oxidative stress precedes amyloid pathology, while mitochondrial dysfunction amplifies this effect through lipid peroxidation and elevated secretase activity [105,106].

### 3.1. Alzheimer’s Disease

In Alzheimer’s disease (AD), deregulation of the MAPK/ERK and Nrf2-ARE pathways contributes to characteristic symptoms such as memory loss, synaptic dysfunction, and tau hyperphosphorylation [107]. Chronic activation of the MAPK/ERK pathway, frequently triggered by amyloid-beta (Aβ) peptides binding to α7 nicotinic acetylcholine receptors, leads to negative consequences such as tau protein hyperphosphorylation, which destabilizes microtubules and impairs neuronal transport [108]. Aberrant signaling leads to neuronal death and increased amyloidogenic processing of APP, resulting in increased Aβ buildup and synaptic dysfunction [109]. Concurrently, the Nrf2-ARE pathway, a critical regulator of cellular antioxidant defenses, is impaired in AD due to dysregulation of its inhibitor Kelch-like ECH-Associated Protein 1 (Keap1), resulting in reduced nuclear translocation of Nrf2 and decreased expression of vital detoxifying enzymes such as Heme Oxygenase-1 (HO-1) and NAD(P)H Quinone Oxidoreductase 1 (NQO) [110]. This failure to establish an efficient antioxidant response permits oxidative stress and neuroinflammation to worsen, harming both astrocytes and neurons and inducing ferroptosis, a type of iron-dependent cell death that exacerbates neuronal loss [111]. The interplay between damaged pathways causes a vicious cycle where Aβ accumulation, tau pathology, and oxidative stress feed into one another, gradually reducing neuronal resilience and ultimately leading to the hallmark cognitive deficits of Alzheimer’s disease [112].

### 3.2. Parkinson’s Disease

Parkinson’s disease (PD) is characterized by progressive neuronal loss in the substantia nigra and the accumulation of Lewy bodies—abnormal protein aggregates within neuronal cytoplasm [113]. Pathologically, this process involves neuronal loss in the substantia nigra and the presence of Lewy bodies, which are abnormal protein deposits in the neuron cytoplasm [114]. Early studies of PD showed that the neurotoxin 1-Methyl-4-PhenylPyridinium (MPP^+^) inhibits mitochondrial complex I, linking disease onset [115]. Mutations in the *parkin* gene, which encodes a ubiquitin E3 ligase, disrupt the proteasome system and impair protein clearance [116]. Parkin normally protects neurons by regulating mitochondrial function and inhibiting apoptotic pathways, but its mutation diminishes this protective role [117].

The Parkinsonism-associated deglycase (DJ-1) protein protects neurons against oxidative stress-induced damage in Parkinson’s disease. Mutations in the *DJ-1* gene impair this defense, increasing neuronal vulnerability [100,118].

### 3.3. Multiple Sclerosis

Multiple sclerosis (MS) is characterized by neuroinflammatory demyelinating lesions and neuronal loss, often driven by progressive disruption of the Blood-Brain Barrier (BBB) [119]. This barrier breakdown allows infiltration of natural killer cells, T and B lymphocytes, and dendritic cells into the spinal cord and brain during MS onset [120]. Epigenetic modifications, particularly DNA methylation, have been observed in immune cells and brain tissues of MS patients, although their precise role in this disease remains unclear [121]. Cellular senescence, which is characterized by the accumulation of lipofuscin in neurons and glial cells within demyelinated lesions across white and gray matter, is proposed to be a significant contributor to progressive MS [122].

The etiology of MS involves a complex interplay between genetic susceptibility and environmental factors, although exact mechanisms remain elusive [123]. These risk factors are believed to synergistically trigger autoimmunity in susceptible individuals [124]. The Antigen-Presenting Cells (APCs) present myelin-derived peptides using class II Major Histocompatibility Complex (MHC) receptors within the central nervous system, activating autoreactive CD4^+^ T lymphocytes [125]. This interaction induces the differentiation of naive CD4^+^ T cells into proinflammatory Th1 cells, which secrete cytokines such as interferon-gamma (IFN-γ), attracting CD8^+^ T cells, B cells, and monocytes to the peripheral regions [126]. These proinflammatory cells then migrate to the BBB, attaching to endothelial cells and intensifying the neuroinflammatory response in MS [123].

### 3.4. Amyotrophic Lateral Sclerosis

Amyotrophic lateral sclerosis (ALS) is a deadly neurodegenerative disease characterized by the gradual loss of both upper and lower motor neurons, which causes paralysis [127]. Early symptoms often include muscle twitching (fasciculations), cramping, stiffness, and weakness in the limbs or bulbar muscles, causing difficulties with walking, speaking, swallowing, and eventually breathing [128]. As the disease advances, muscle paralysis spreads throughout the body, severely impairing mobility and respiratory function [129]. Despite these physical declines, ALS typically does not affect a person’s intelligence or sensory functions, although some patients may experience cognitive or behavioral changes, including frontotemporal dementia in a subset of cases [130].

Its pathophysiology is complicated, including genetic abnormalities, immunological dysregulation, oxidative stress, and altered protein and RNA metabolism [131]. Mutations in these genes disturb protein homeostasis by disrupting the ubiquitin-proteasome system and autophagy, resulting in toxic protein aggregation and neuronal death [132]. When the RNA-binding proteins TDP-43 and FUS are mislocalized or aggregated, they lead to aberrant RNA metabolism [133]. Recent research has also identified genetic variations in genes such as *CNTN4*, *DPP6*, and *INPP5B* that may influence ALS susceptibility. These coupled circuits form a complicated disease process that gradually compromises motor neuron function.

### 3.5. Huntington’s Disease

As detailed in Section 3, Huntington’s Disease (HD) involves disrupted cellular processes including mitochondrial dysfunction, protein misfolding, and excitotoxicity, which together compromise neuronal function and survival [128,129]. The huntingtin protein is ubiquitously expressed in human and animal cells, with relatively high levels in the brain and testes, and moderate levels in the liver, heart, and lungs [134]. Orthologues of this protein are found in various species, including zebrafish, fruit flies, and slime molds [135].

Cognitive decline is another significant feature of HD, affecting memory, executive functions, and decision-making abilities. Psychiatric disturbances such as depression, anxiety, irritability, and personality changes are also commonly observed [136]. Disruptions in protein clearance mechanisms, including autophagy and the ubiquitin-proteasome system, contribute to the buildup of toxic protein aggregates [137]. Excitotoxicity from neurotransmitter imbalance worsens neuronal damage [138].

Dysregulation of intracellular signaling pathways involving calcium, cAMP Response Element-Binding (CREB) protein, and Brain-Derived Neurotrophic Factor (BDNF) also contributes to the pathophysiology of HD [139]. Although haploinsufficiency—a deficiency in protein production due to genetic factors—has been proposed in autosomal-dominant diseases, it is unlikely to explain HD since partial loss of the HD gene does not cause the disease phenotype in humans [139]. In cell culture models, HD is associated with the activation of proapoptotic enzymes such as caspase 1 or 8, contributing to polyglutamine toxicity [140]. Molecular chaperones, such as Hsp 40 and 70, play crucial roles in protein folding and have been shown to mitigate the aggregation and toxicity of polyglutamine-containing proteins [141].

### 3.6. Creutzfeldt-Jakob Disease

Diagnosis of Creutzfeldt-Jakob Disease (CJD) is often delayed due to its rarity and low clinical suspicion during a patient’s lifetime [142]. The symptoms of CJD typically include rapidly progressive dementia, neurological disturbances, and involuntary movements [143]. As the disease progresses, individuals may experience severe cognitive impairment, muscle stiffness, myoclonus (sudden jerking movements), and difficulties with coordination and balance. Other symptoms can include changes in mood and behavior, hallucinations, and sensory abnormalities [144].

The exact cause of prion diseases, such as CJD, is not fully understood [145]. Genetic factors or environmental exposure are not typically associated with prion diseases. Instead, it is believed that a stochastic misfolding event of the normal prion protein (PrPC) or a somatic mutation in the *PRNP* gene within a single or clade of cells may trigger the disease [146]. Prion diseases are transmissible, and cases of acquired variants have been documented. One notable example is the outbreak of “kuru”, which affected the Fore ethnic group and neighboring communities in Papua New Guinea [147]. This outbreak, which occurred due to ritual cannibalism, resulted in over 3000 cases [147]. More recently, variant CJD (vCJD) emerged as a zoonotic outbreak with over 230 confirmed cases caused by the dietary transfer of BSE prions [148].

### 3.7. Perinatal Stroke

Perinatal Stroke (PS) is a complex condition that causes brain damage at a key developmental phase [147]. The growing brains of neonates have amazing flexibility, allowing them to use alternate neural pathways and perhaps achieve unexpected beneficial results despite the harm sustained [148]. A medical history, physical examination, and imaging methods, such as Magnetic Resonance Imaging (MRI) or computed tomography (CT), are usually used to diagnose the brain and discover abnormalities or indicators of damage. PS refers to six unique prenatal stroke syndromes classified by vascular involvement, stroke mechanism, time of damage, and clinical presentation [149].

Acute presentations include Newborn Arterial Ischemic Stroke (NAIS), Neonatal Cerebral Sinovenous Thrombosis (CSVT), and Neonatal Hemorrhagic Stroke (NHS). Delayed presentations include Presumptive Prenatal Hemorrhagic Stroke (PPHS), Periventricular Venous Infarction (PVI), and Arterial Presumed Perinatal Ischemic Stroke (APPIS) [150]. Treatment for PS primarily focuses on managing immediate complications and providing supportive care [151]. This may involve medications to prevent seizures or manage other medical issues, as well as rehabilitation therapies such as physical therapy or occupational therapy to promote optimal development [152].

### 3.8. Duchenne Muscular Dystrophy

Duchenne Muscular Dystrophy (DMD) frequently begins with delayed motor milestones in early childhood [149]. Children with DMD often exhibit an abnormal, waddling gait and experience frequent falls [153]. As the disease progresses, muscle weakness and wasting become more pronounced, making activities requiring muscle strength, such as climbing stairs or lifting objects, increasingly difficult [154].

The diagnosis of DMD typically involves genetic testing to identify mutations or deletions in the *DMD* gene [155]. Elevated levels of the enzyme Creatine Kinase (CK) in the blood can also indicate muscle damage, supporting the diagnostic process [156]. Muscle biopsies may be performed to assess dystrophin levels and evaluate muscle pathology.

The pathophysiology of DMD involves the absence or deficiency of dystrophin, which disrupts the structural stability of muscle fibers [157]. Dystrophin is part of the Dystrophin-associated Glycoprotein Complex (DGC), which links the internal cytoskeleton of muscle cells to the extracellular matrix [158]. This linkage is crucial for maintaining muscle cell membrane integrity during contraction and relaxation [158]. Without dystrophin, muscle fibers are more susceptible to damage, leading to progressive muscle degeneration and replacement with fibrotic tissue [159].

Table 4 highlights key mechanistic pathways and reported therapeutic effects of *U. dioica* across models.

## 4. Effects of *Urtica dioica* on Neurodegenerative Disorders in Animal Models

**Figure 6:**
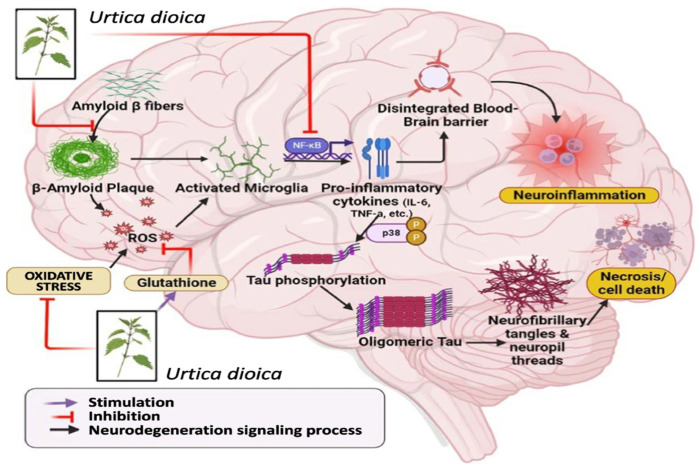
Mechanisms of neuroprotection associated with *Urtica dioica*. Reprinted with permission from [34].

New experimental research in a variety of preclinical settings supports *U. dioica’s* neurotherapeutic potential [176]. Its activities seem to converge on a number of fundamental protective pathways rather than separate outcomes, most notably redox homeostasis, anti-inflammatory signaling, and neurotrophin control —as illustrated in Figure 6 [177]. In zebrafish neurotoxicity studies, ethanolic *U. dioica* extract showed neuroprotection against chlorpyrifos-induced damage by reducing abnormalities, preserving forebrain shape, and restoring locomotor function [29].

Diabetic rat models, frequently employed as a proxy for neurodegenerative progression, improved with *U. dioica* (50–100 mg/kg), displaying recovered memory performance and endocrine balance, rivaling established treatments like rosiglitazone [178]. Additionally, hydroalcoholic preparations demonstrated histological and molecular advantages in the hippocampus of streptozotocin-induced rats, indicating Nerve Growth Factor (NGF) and BDNF regulation [12]. In cholinergic mice, *U. dioica* leaf extract not only corrected scopolamine-induced cognitive loss [70], but it also increased the efficiency of co-administered minocycline, indicating synergistic neuroprotection [179]. A separate line of research confirms that therapy with *U. dioica* reduces acetylcholinesterase activity, lowers oxidative stress indicators, and improves memory learning [7]. These effects correspond to increased transcription of genes associated with sporadic Alzheimer’s disease, providing mechanistic evidence for its functional results.

## 5. Conclusions

*Urtica dioica* has been utilized for decades, serving as both a nutritional resource and a traditional medicinal remedy. The neuroprotective effectiveness of medicinal plants can be attributed to their ability to exhibit diverse mechanisms, including antioxidant activity and inflammation inhibition. The promising outcomes of these studies suggest that *U. dioica*, with its diverse phytochemical composition, has the potential to manage neuroinflammation, oxidative stress, and mitochondrial dysfunction [179,180].

While previous research has provided insights into its anti-neurological properties, more comprehensive studies are needed to pinpoint the specific signaling pathways affected by *U. dioica*. This identification could potentially facilitate the development of future drugs for treating neurodegenerative disorders. Additionally, integrating Artificial Intelligence (AI) into *U. dioica* research offers a promising pathway to accelerate discoveries. AI-driven data analysis and predictive modeling can identify key bioactive compounds and their mechanisms, optimize experimental protocols, and enhance precision in biomarker identification. Such innovative approaches could deepen our understanding of *U. dioica* and pave the way for its personalized and clinical applications in managing neurodegenerative diseases.

## Figures and Tables

**Table 1:** Phytochemicals found in *Urtica dioica* leaves.

Phytochemicals [References]	Chira et al. 2022 [8]	Ðurovic et al. 2024 [38]	Koczkoday et al. 2023 [39]	Yousuf et al. 2022 [40]	Brahmi-Chendouh et al. 2021 [41]	Ðurovic et al. 2023 [42]
Protocatechuic acid [38,42]	n.d.	**X**	n.d.	n.d.	n.d.	**X**
p-Hydroxybenzoic acid [38,41]	n.d.	**X**	n.d.	n.d.	**X**	n.d.
Caffeic acid [38,41,42]	n.d.	**X**	n.d.	n.d.	**X**	**X**
Vanillic acid [38,42]	n.d.	**X**	n.d.	n.d.	n.d.	**X**
Aesculin [38]	n.d.	**X**	n.d.	n.d.	n.d.	n.d.
5-O-Caffeoylquinic acid [38,39]	n.d.	**X**	**X**	n.d.	n.d.	n.d.
p-Coumaric acid [38,41,42]	n.d.	**X**	n.d.	n.d.	**X**	**X**
Ferulic acid [38,42]	n.d.	**X**	n.d.	n.d.	n.d.	**X**
p-Hydroxyphenylacetic [38]	n.d.	**X**	n.d.	n.d.	n.d.	N.d.
Quercetin-3-O-galactoside [38,39]	n.d.	**X**	**X**	n.d.	n.d.	N.d.
Quercetin-3-O-rutinoside (Rutin) [38,39,40,42]	n.d.	**X**	**X**	**X**	n.d.	**X**
Apigenin-7-O-glucoside [38]	n.d.	**X**	n.d.	n.d.	n.d.	n.d.
Quercetin [38,40,42]	n.d.	**X**	n.d.	**X**	n.d.	**X**
Luteolin [38,41]	n.d.	**X**	n.d.	n.d.	**X**	n.d.
Naringin [38]	n.d.	**X**	n.d.	n.d.	n.d.	n.d.
Naringenin [38,42]	n.d.	**X**	n.d.	n.d.	n.d.	**X**
Kaempferol [38,42]	n.d.	**X**	n.d.	n.d.	n.d.	**X**
Kaempferol 3-O-glucoside [42]	n.d.	n.d.	n.d.	n.d.	n.d.	**X**
Apigenin [38,41]	n.d.	**X**	n.d.	n.d.	**X**	n.d.
Isorhamnetin-3-O-rutinoside [38]	n.d.	**X**	n.d.	n.d.	n.d.	n.d.
Taxifolin [38]	n.d.	**X**	n.d.	n.d.	n.d.	n.d.
Isorhamnetin-3-O-glucoside [38]	n.d.	**X**	n.d.	n.d.	n.d.	n.d.
Daidzein [38]	n.d.	**X**	n.d.	n.d.	n.d.	n.d.
Eriodictyol [38]	n.d.	**X**	n.d.	n.d.	n.d.	n.d.
Chrysoeriol [38]	n.d.	**X**	n.d.	n.d.	n.d.	n.d.
Chrysin [38]	n.d.	**X**	n.d.	n.d.	n.d.	n.d.
Acacetin [38]	n.d.	**X**	n.d.	n.d.	n.d.	n.d.
Genkwanin [38]	n.d.	**X**	n.d.	n.d.	n.d.	n.d.
Galangin [38]	n.d.	**X**	n.d.	n.d.	n.d.	n.d.
Kaempferide [38]	n.d.	**X**	n.d.	n.d.	n.d.	n.d.
Cinnamic acid [42]	n.d.	n.d.	n.d.	n.d.	n.d.	**X**
Syringic acid [42]	n.d.	n.d.	n.d.	n.d.	n.d.	**X**
Chlorogenic acid [39,42]	n.d.	n.d.	**X**	n.d.	n.d.	**X**
Neochlorogenic acid [39]	n.d.	n.d.	**X**	n.d.	n.d.	n.d.
Sinapic acid [42]	n.d.	n.d.	n.d.	n.d.	n.d.	**X**
Gallic acid [42]	n.d.	n.d.	n.d.	n.d.	n.d.	**X**
Cichoric acid [39]	n.d.	n.d.	**X**	n.d.	n.d.	n.d.
Hyperoside [39]	n.d.	n.d.	**X**	n.d.	n.d.	n.d.
Rutoside [39]	n.d.	n.d.	**X**	n.d.	n.d.	n.d.
Pentenal [8]	**X**	n.d.	n.d.	n.d.	n.d.	n.d.
1-8-Cineol [8]	**X**	n.d.	n.d.	n.d.	n.d.	n.d.
Cis-linalool oxide [8]	**X**	n.d.	n.d.	n.d.	n.d.	n.d.
Trans-linalool oxide [8]	**X**	n.d.	n.d.	n.d.	n.d.	n.d.
β-linalool [8]	**X**	n.d.	n.d.	n.d.	n.d.	n.d.
Camphor [8]	**X**	n.d.	n.d.	n.d.	n.d.	n.d.
Menthone [8]	**X**	n.d.	n.d.	n.d.	n.d.	n.d.
Cyclohexanone [8]	**X**	n.d.	n.d.	n.d.	n.d.	n.d.
Borneol [8]	**X**	n.d.	n.d.	n.d.	n.d.	n.d.
Menthol [8]	**X**	n.d.	n.d.	n.d.	n.d.	n.d.
α-Terpineol [8]	**X**	n.d.	n.d.	n.d.	n.d.	n.d.
Carvone [8]	**X**	n.d.	n.d.	n.d.	n.d.	n.d.
Borneol-acetate [8]	**X**	n.d.	n.d.	n.d.	n.d.	n.d.
Cariophylène oxide [8]	**X**	n.d.	n.d.	n.d.	n.d.	n.d.
Globulol [8]	**X**	n.d.	n.d.	n.d.	n.d.	n.d.
Ethyl palmitate [8]	**X**	n.d.	n.d.	n.d.	n.d.	n.d.
Phytol [8]	**X**	n.d.	n.d.	n.d.	n.d.	n.d.
Ethyl oleate [8]	**X**	n.d.	n.d.	n.d.	n.d.	n.d.
Cetanol [8]	**X**	n.d.	n.d.	n.d.	n.d.	n.d.
Tetradecanoic acid [8]	**X**	n.d.	n.d.	n.d.	n.d.	n.d.
Eicosanol [8]	**X**	n.d.	n.d.	n.d.	n.d.	n.d.
Eicosene [8]	**X**	n.d.	n.d.	n.d.	n.d.	n.d.
Decanol [8]	**X**	n.d.	n.d.	n.d.	n.d.	n.d.
Loxanol [8]	**X**	n.d.	n.d.	n.d.	n.d.	n.d.
3-Pyridinecarbonitrile [8]	**X**	n.d.	n.d.	n.d.	n.d.	n.d.
3-Ecosene (E) [8]	**X**	n.d.	n.d.	n.d.	n.d.	n.d.
Benzene dicarboxylic acid [8]	**X**	n.d.	n.d.	n.d.	n.d.	n.d.
Oleic acid [8,38,41,42]	**X**	**X**	n.d.	n.d.	**X**	**X**
Diethyl methyl borane [8]	**X**	n.d.	n.d.	n.d.	n.d.	n.d.

n.d., not determined.

**Table 2:** Protective and Therapeutic Effects in Animal and Human Studies of Compounds Isolated from *Urtica dioica*.

Phytochemicals [References]	Animal Model or Human Study	Protective and Therapeutic Effects
Flavonoids (rutin, isoquercetin, kaempferol-3-O-rutinoside, isorhamnetin-3-O-rutinoside, kaempferol-3-O-glucoside, isorhamnetin-3-O-glucoside) [49]	Rat surgical endometriosis model	Significantly reduces implant volumes and adhesion scores of endometriotic implants. Decreases TNF-α, VEGF, and IL-6 levels in peritoneal fluids. Histopathological findings support the biological activity results.
Caffeoylmalic acid [50]	Mouse diabetes model	Exhibits antidiabetic activity by stimulating glucose absorption and reducing blood glucose levels. Ameliorates lipid profile, liver, and blood parameters, with a moderate effect on insulin secretion. Inhibits α-amylase and α-glucosidase activities.
2,4-di-t-butylphenol, neophytadiene, butyl tetradecyl ester, dibutyl phthalate, bis(2-ethyl hexyl) maleate, 1,2-benzenedicarboxylic acid, 2-t-butyl-4,6-bis(3,5-di-t-butyl-4-hydroxybenzyl)phenol [51]	*Wistar* rats	Exhibits anti-inflammatory activity (part of fraction-II) with 48.83% inhibition at 200 mg/kg bw/*pro die*, comparable to indomethacin. Shows anti-microbial activity against various bacterial strains (e.g., *Enterococcus faecalis*, *Escherichia coli*, *Klebsiella pneumoniae*, *Pseudomonas aeruginosa*, *Staphylococcus aureus*, *Shigella flexneri*, and *Salmonella typhi*).
Vitexin (apigenin-8C-glucoside), chlorogenic acid, caffeic acid, vicenin-2 (6,8-di-C-glucosyl apigenin) [52]	Mice (writhing, formalin, and hot plate tests)	Produced significant inhibition on nociception induced by acetic acid and formalin: at a dose of 10 mg/kg bw/*pro die*, intraperitoneally: vitexin showed 91% inhibition, chlorogenic acid 72%, caffeic acid 41%, and vicenin-2 41%. The activation of cholinergic systems seems to be involved in the mechanism of antinociception
Phenolic acids (5-O-caffeoylquinic acid), Flavonol glycosides (rutin, isoquercitrin, kaempferol 3-O-glucoside), Lignans (secoisolariciresinol, 9,9′-bisacetyl-neo-olivil and their glucosides) [53]	Human platelets Intestinal epithelial cells	Inhibit cyclooxygenase and lipoxygenase pathways. Herb extracts inhibit 12-lipoxygenase pathway; root extracts inhibit thromboxane production. Increase monocyte chemoattractant protein-1 and growth-related oncogene release, stimulating MyD88/NF-κB/p38 signaling, preserving epithelial integrity and enhancing intestinal defense mechanisms. Root extract reduces lipopolysaccharide-induced monocyte chemoattractant protein-1/growth-related oncogene secretion and cyclooxygenase-2 expression, showing potential protective effect against tissue damage caused by inflammation.
Polyphenols (e.g., flavonoids, phenolic acids) [42]	Human breast cancer cell lines BALB/c mice	Exhibits anti-proliferative and apoptotic effects on various human cancers, including breast cancer. Inhibits cancer cell growth, induces apoptosis, and reduces tumor volume. Enhances sensitivity to chemotherapy drugs like paclitaxel and cisplatin. Possesses anti-oxidant, anti-mutagenic, and anti-proliferative properties.
Pioglitazone (PIO) [54]	Streptozotocin (STZ)-induced diabetic mice	Reduces oxidative stress and hyperglycemia; alleviates neurotoxicity.
Vitamin E [55]	Streptozotocin (STZ)-induced diabetic mice	Reduces oxidative stress and ameliorates pain.
Quercetin [56]	Mouse (Chronic Unpredicted Stress Model)	Reduces anxiety. Attenuates depression. Improves cognitive dysfunction. Normalizes locomotor activity. Lowers oxidative stress markers. Enhances antioxidant levels. Reduces pro-inflammatory cytokines. Prevents hippocampal neuronal damage.
Ferulic acid [57]	Rats	Antioxidant potential: ferulic acid, isolated from the ethyl acetate fraction (EAF) of *U. dioica*, showed potent antioxidant properties, reducing oxidative stress in both in-vitro and in-vivo models. Hepatoprotective potential: ferulic acid significantly attenuated increased liver enzymes and oxidative parameters in CCl4-induced hepatotoxicity, demonstrating its protective effect on the liver.
β-Sitosterol [58]	Rats	Significant reduction in prostate/body weight ratio. Decreased serum testosterone levels. Lowered prostate-specific antigen levels. Improved histological examinations of the prostate.
Urticol [59]	Ex vivo assays using primary rat hepatocytes	Reduced elevated glucose levels by stimulating glucose uptake by 28.57% compared to untreated control and by 11.45% compared to pioglitazone (100 μM) (*p* ≤ 0.05). Shows potential as an antihyperglycemic agent, possibly through the induction of Glut-4 expression in hepatocytes.
Histamine H3R-antagonists (ciproxifan, clobenpropit) [60]	Animal models of schizophrenia (SCZ)	Controlled elevated oxidative stress markers: TBARS, GSH, superoxide dismutase, catalase. Prevented oxidative stress. Alleviated schizophrenic symptoms, focusing on negative symptoms and cognitive deficits. Exhibited antioxidant activity. Supplemented antioxidant needs in SCZ.

**Table 3:** Summary of the Potential Neuroprotective Effects of *Urtica dioica* L. Phytochemicals from Different Regions.

Phytochemicals [References]	Antioxidant Potential	Anti-Inflammatory Potential	Neuroplasticity Potential	Anti-Neuropathological Protein Accumulation Potential
Caffeic acid derivative [61,62,63]	Reduced levels of reactive oxygen species (ROS) and lipid peroxidation (LPO).	Decreased expression of inflammatory markers (Iba-1, GFAP).	Increased expression of synaptic proteins SNAP-25 and PSD-95, improving synaptic function.	Inhibited the formation of α-synuclein oligomers, prevented Aβ peptide fibrillation and oligomerization, and enhanced expression of Nrf2 and HO-1.
Carvone [64]	Significant increase in both enzymatic and non-enzymatic antioxidants in Freund’s Complete Adjuvant-induced arthritic rats.	Immunostimulating and immunosuppressive effects depending on mouse model.	Improved memory in BALB/c mice; altered memory in C57BL/6J mice.	Inhibits butyrylcholinesterase (40%).
Quercetin [56,65,66]	High ROS scavenging capability.	Reduce the production of inflammatory cytokines including IL-6 and IL-1.	Increased expression of BDNF, p-CREB, and FoxG1 in DG promoting AHN.	Inhibition of mTORC1, reducing β-amyloid, α-synuclein, or huntingtin levels.
Acetylcholine [67,68]	Regulation of the activity of antioxidant enzymes (ascorbate peroxidase, catalase).	Inhibiting production of proinflammatory cytokines (ILs, TNF-α, HMGB-1).	Prevent neuronal apoptosis (increase cleaved caspase 3 induced by IFN-β).	High affinity for synuclein binding in Parkinson’s disease patients.
Histamine [60,69]	Stimulation of immune cells, such as neutrophils, leads to ROS generation.	Inhibit prostaglandin production via COX-1, COX-2, HPGDS to prevent degranulation.	Modulation of protein synthesis-dependent synaptic plasticity via histamine and NMDA receptors.	Preservation of CREB and PSD-95 essential for neuronal and synaptic function.
Serotonin [70,71]	Elevated serotonin levels improve microcirculation and tissue recovery.	Reduction of pro-inflammatory cytokines (COX-2, IL-6).	Enhanced BDNF, VEGF, arc expression, and increased neurogenesis in the hippocampus.	Modulation of cortical Tau and hippocampal Hsp70, activation of BDNF/TrkB/CREB pathway in neuronal cells.
Phylloxanthobilin (PxB) [72,73]	Demonstrated the strongest antioxidative properties in cell-free and cell-based systems among tested phyllobilins.	PxB inhibits the catabolism of tryptophan to kynurenine in human peripheral blood mononuclear cells, indicating a suppressive effect on immune activation pathways.	PxB enhances synaptic plasticity, crucial for learning and memory, by inhibiting amyloid β (Aβ) aggregation, a hallmark of AD.	PxB’s ability to modulate immune responses may reduce neurotoxic protein accumulation, mitigating neurodegeneration.
Lutein [61,74,75]	Scavenges ROS (singlet oxygen, lipid peroxy radicals), reducing oxidative stress linked to metabolic diseases.	Modulates NF-κB, suppressing pro-inflammatory cytokines (IL-1β, TNF-α), preventing chronic inflammation.	Enhances synaptic proteins expression, supporting neuroprotection and reducing cognitive decline.	Mitigates aggregation of misfolded proteins in neurodegenerative conditions through antioxidant activity.
β-carotene [72,73,74]	Exhibits antioxidant properties with lower bioaccessibility (~10%).	Reduction of iNOS and COX-2 expression in LPS-stimulated macrophages decreases inflammatory mediators.	Enhances synaptic integrity, facilitating LTP and LTD, essential for learning and memory.	Inhibits Aβ peptide aggregation, reducing amyloid fibril formation and stabilizing soluble Aβ forms to prevent toxic plaque formation.
Phenolic acids [53,76,77,78,79]	Promotes Nrf2 nuclear translocation, enhancing antioxidant defense genes like heme oxygenase and NAD(P)H quinone oxidoreductase 1 (NQO1).	Selective inhibition of cyclooxygenase and lipoxygenase pathways.	Inhibits monoamine oxidase (MAO) enzymes, improving neurotransmitter availability, neuroplasticity, and reducing depressive-like behavior.	Enhances clearance of misfolded proteins via autophagy and the ubiquitin-proteasome system, preventing toxic effects of protein accumulation.
Flavonol glycosides [53,80]	Eliminate hydroxyl radicals generated from oxidative stress.	Inhibits cyclooxygenase and lipoxygenase pathways.	Stimulates neurite and neuronal synapse formation, enhanced by treatments like RhoA inhibitors.	Promotes misfolded protein clearance through autophagy and ubiquitin-proteasome system, maintaining protein homeostasis.
Lignans (e.g., secoisolariciresinol, 9,9′-bisacetyl-neo-olivil, 7-hydroxymatairesinol) [53,81,82]	Inhibits lipid peroxidation and eliminates hydroxyl radicals.	Better inhibition of thromboxane production; reduced inflammatory markers.	Protects against neurodegeneration, alleviates memory impairment in Parkinson’s disease models.	Promotes neurotrophic factor expression (e.g., BDNF), enhances misfolded protein clearance, and improves neuronal resilience.

**Table 4:** Comparative Summary of Disease Mechanisms and *Urtica dioica* Effects in Neurodegenerative Models.

Disease	Core Pathogenic Mechanisms	Experimental Effects of *U. dioica*
Alzheimer’s Disease (AD)	Tau pathology, oxidative stress dysfunction, and Aβ accumulation [160]	Enhances memory and lessens cholinergic and oxidative stress in animal models of diabetes and scopolamine [70]
Parkinson’s Disease (PD)	α-synuclein aggregation, mitochondrial damage, and reduced protein clearance [161]	Enhances antioxidant enzyme activity and reduces neuroinflammation (TNF-α/IL-1β levels) in MPTP mouse models [34]
Multiple Sclerosis (MS)	Immune cell infiltration, ROS-induced demyelination, and axonal deterioration [162]	Reduces Heat Shock Protein 60 (HSP60) expression and promotes neuroregeneration (Experimental Autoimmune Encephalomyelitis) [10]
Amyotrophic Lateral Sclerosis (ALS)	Genetic mutations (SOD1, TDP-43, FUS, C9orf72), protein aggregation, mitochondrial dysfunction, excitotoxicity, RNA metabolism defects, and neuroinflammation [163]	Flavonoid component (rutin) in *U. dioica* improves motor outcomes and reduces protein aggregation in SOD1-G93A models [164,165]
Huntington’s Disease (HD)	Mutant huntingtin (m*HTT*) aggregation, CAG repeat expansion, and striatal neuron degeneration [166,167,168]	No direct models tested [166]. However, omega-3 fatty acids (e.g., DHA and EPA) in *U. dioica* may counteract mitochondrial malfunction and excitotoxic brain damage, both significant in HD development [134,135]
Perinatal Stroke (PS)	Ischemia or hemorrhage, impaired neuronal development, long-term motor and cognitive deficits [169]	Pretreatment with extracts of *Viola spathulata*, *U. dioica*, and *Lamium album* significantly decreased XBP-1 splicing in stroke rats’ brains, indicating ER stress reduction [170]. Specifically, in groups treated with *U. dioica*, XBP-1 gene splicing was significantly inhibited, even totally halted in healthy rats given *U. dioica* alone [171]
Creutzfeldt-Jakob Disease (CJD)	Prion proteins aggregation, oxidative stress, rapid neurodegeneration [172,173]	No experimental data [45]; antioxidative properties suggest potential relevance may be related to the pathophysiology of neurodegeneration, including those found in CJD [174]
Duchenne Muscular Dystrophy (DMD)	Dystrophin loss destabilizes muscle fibers, facilitates calcium influx, and triggers oxidative and immune stress [175]	No direct studies [45]; neuroprotective, anti-inflammatory, antioxidant, and metabolic benefits supports theoretical applicability [34]

## Data Availability

Not applicable.
